# Manual lymphatic drainage therapy in patients with breast cancer related lymphoedema

**DOI:** 10.1186/1471-2407-11-94

**Published:** 2011-03-09

**Authors:** Marta López Martín, Miguel A Hernández, Cristina Avendaño, Francisco Rodríguez, Helena Martínez

**Affiliations:** 1 Unidad de Investigación, Hospital Universitario de La Princesa, Diego de León 36, 28046 Madrid, Spain; 2 Fundación Laín Entralgo, Gran Vía 27, 2813 Madrid, Spain

## Abstract

**Background:**

Lymphoedema is a common and troublesome condition that develops following breast cancer treatment. The aim of this study is to analyze the effectiveness of Manual Lymphatic Drainage in the treatment of postmastectomy lymphoedema in order to reduce the volume of lymphoedema and evaluate the improvement of the concomitant symptomatology.

**Methods:**

A randomized, controlled clinical trial in 58 women with post-mastectomy lymphoedema. The control group includes 29 patients with standard treatment (skin care, exercise and compression measures, bandages for one month and, subsequently, compression garnments). The experimental group includes 29 patients with standard treatment plus Manual Lymphatic Drainage. The therapy will be administered daily for four weeks and the patient's condition will be assessed one, three and six months after treatment.

The primary outcome parameter is volume reduction of the affected arm after treatment, expressed as a percentage. Secondary outcome parameters include: duration of lymphoedema reduction and improvement of the concomitant symptomatology (degree of pain, sensation of swelling and functional limitation in the affected extremity, subjective feeling of being physically less atractive and less feminine, difficulty looking at oneself naked and dissatisfaction with the corporal image).

**Discussion:**

The results of this study will provide information on the effectiveness of Manual Lymphatic Drainage and its impact on the quality of life and physical limitations of these patients.

**Trial registration:**

ClinicalTrials (NCT): NCT01152099

## Background

Nowadays, breast cancer is the most frequent malignancy in women, with an incidence of 35-44 new cases per 100.000 women/per year, and its frequency is increasing. Approximately 25% of breast cancer patients develop lymphoedema following breast cancer treatment, and the risk increases every year [1]. Thus, breast cancer-related lymphoedema is one of the main complications and most dreaded sequela of breast cancer and its therapies, and can have long-term physical and psychosocial consequences for patients. It consists of the accumulation of lymph in the interstitial spaces, principally in the subcutaneous fatty tissues, caused by a defect in the lymphatic system. It is marked by an abnormal increase of tissue proteins, oedema, chronic inflammation and fibrosis [2]. Three stages of lymphoedema have been described. Stage I presents with pitting and is considered reversible; some women in this stage have no increased arm girth or heaviness and no signs of pitting oedema. As the oedema progresses, it becomes brawny, fibrotic, non-pitting and irreversible (stage II). In advanced lymphoedema (stage III), which rarely occurs following breast cancer treatments, cartilaginous hardening occurs, with papillomatous outgrowths and hyperkeratosis of the skin. Problems associated with lymphoedema include: pain, altered sensations such as discomfort and heaviness, difficulties with physical mobility, physiological distress, recurrent infections and social isolation [3,4]. Lymphoedema has been shown to significantly negatively affect quality of life, [5] and it is a traumatic condition because apart from the inconveniences and functional impairment that starts at the arm level, the aesthetic factor plays a determinant role in the loss of self-esteem, which causes psychological sequelae in many patients [6]. Some quality of life scales are available to determine how the affected patients define, admit and explain their personal experience regarding the symptoms they experience and in which way these symptoms have an influence on the degree of functionality regarding daily activities and interpersonal relationships. In 1980 The European Organization for Research and Treatment of Cancer (EORT) created a group to study the quality of life in patients with cancer and developed the EORT Quality of Life Questionnaire (EORT QLQ-C30). Subsequently EORT QLQ-BR 23 was developed to assess the specific aspects of lymphoedema in patients with breast cancer. The latter evaluates the influence of surgery, chemotherapy, radiotherapy and hormone therapy on the homolateral arm of the treated breast, the corporal image, sexuality, perspectives of future, symptoms of the disease and function of the upper extremity, evaluating a total of 23 items. Both questionnaires are validated for the Spanish population [7]. In a study in which 744 patients with breast cancer were assessed with these quality of life scales, some imbalances regarding the quality of life in those patients with lymphoedema were found [8]. Definitively, lymphoedema is one of the most severe sequelae that affects health-related quality of life in treated breast cancer patients.

Lymphoedema treatment remains a problem even with modern treatment modalities, since clear therapeutic protocols do not exist. Several methods have been used with varying results. Among them the physical treatment introduced by Winiwarter (1892), improved by Vodder (1932-1960) and later named Complex Physical Therapy (CPT) by Foldie, has been recommended by several groups of experts [9]. Complex Physical Therapy, also called Complex Decongestive Physiotherapy, is a treatment regimen that includes meticulous skin hygiene, manual lymph drainage, bandaging, exercises and supportive garments. This therapy is carried out in two phases; in the first phase (treatment), the aim is to mobilize the lymph accumulated, reduce the fibrous tissue and improve the health of the skin using mainly daily Manual Lymph Drainage (MLD) during a variable period of time. In addition, patients receive instructions regarding skin care, prophylactic measures and the use of multilayer bandages. In the second phase (maintenance), compression bandaging, regular physical exercise and weight control are used [10].

Although the first results have been optimistic, in randomized controlled clinical trials, the CPT has not clearly demonstrated any superiority when compared with other simpler alternatives and no study has evaluated patients' treatment preferences or the effects of the treatment on quality of life [11]. Several authors suggest that this technique should be reserved only for selected cases. The proof of concept for using CPT to stimulate the lymph drainage has a profound physiological basis, but the quality of evidence on the relative effectiveness of therapies is poor. On the other hand, the fact that CPT implies several techniques (manual lymph drainage, skin care and multilayer compression bandaging followed by a compression garment to reduce oedema and therapeutic exercises) makes it difficult to recognize which of them is the truly effective one in the treatment of lymphoedema [12-14].

Manual Lymph Drainage (MLD) is a massage technique that involves the skin surface only and follows the anatomic lymphatic pathways of the body. A session of manual lymph drainage starts centrally in the neck and trunk to clear out the main lymphatic pathways, thereby facilitating drainage from the arm. Unlike other types of massage, it produces neither blush nor pain and it does not even have a stimulant effect. To successfully complete this technique, a knowledge of the anatomical distribution of the lymphatic and superficial ganglions and of their interconnections is necessary. The MLD is carried out in a descending manner to facilitate the flow of the lymph from the affected areas to those not affected. It is possible to carry out MLD in the case of late or latent lymphoedema three days postsurgery if contraindications do not exist. There are several schools (Vodder, Leduc, Foldie), but they all agree on the fundamental aspects. The MLD must be applied by a physical therapist trained for it. Its application demands a long period of time (from 45 to 60 min.) and it is usually performed once a day (four or five times a week) for 2 to 4 weeks. A study carried out by Leduc and Colls demonstrated that the greatest reduction of the oedema is obtained in the first week of treatment; during the second week the results became stable [15].

The results from several case studies (including over 400 patients) clearly showed that treatment with MLD and compression bandaging had a volume-reducing effect. However, the interpretation of these results is limited due to the methodology used, and their design did not offer the possibility to investigate the extent to which MLD treatment contributed to the reduction of lymphoedema.

Only three small, randomized, controlled trials have assessed MLD for arm lymphoedema but contradictory results have been found. Two of the studies showed statistically significant differences in the reduction of oedema that favored the group on combined treatment with MLD, despite the fact that in one of the studies, MLD was only administered for one week. The first study, conducted in 35 women, compared compression bandaging plus MLD with compression bandaging alone (level III evidence) [16]. There was a trend in mean volume reduction and a statistically significant difference between the 2 groups in the percentage reduction in volume in favour of the combined treatment. In the second trial, Manual Lymph Drainage plus a compression garment use was compared with sequential pneumatic compression plus compression garment use; no difference was detected between the treatment groups (level II evidence) [17].

The third published randomized trial, involving 42 women with modest stage I or II lymphoedema compared standard therapy alone with standard therapy plus MLD and training in self-massage (level I evidence). Standard therapy included the use of a custom-made sleeve-and-glove compression garment worn during the day, instruction in physical exercises, education in skin care, and information and recommendations about lymphoedema. Both groups obtained a significant reduction in limb volume, a decrease in discomfort and increased joint mobility over time. However, no significant differences in objective measures regarding change in arm volume or subjective measures of symptoms related to lymphoedema were found between the 2 groups [18]. This study investigated the effect of eight sessions of MLD over two weeks, so the MLD course was relatively short and the study group was limited to those with mild to moderate swelling (20-30% of difference). Information about quality of life was gathered using the questionnaire EORT QLQ-C30, but the results were not considered in the final evaluation. On the other hand, there is no evidence that shows that the effect of MLD is permanent - because previous studies measured the volume directly after the conclusion of MLD - or if the MLD can be effective in decreasing the symptoms, in spite of not obtaining an improvement in volume reduction. Therefore, the utility of MLD as part of the Physical Complex Therapy has turn out to be relevant, given the exclusive dedication of the therapist that it entails and the extra cost in relation to the potential health benefits for patients.

The aim of this study is to analyze the short-term and long-term effectiveness of Manual Lymphatic Drainage (MLD) in the treatment of postmastectomy lymphoedema in order to reduce the volume of lymphoedema and evaluate the improvement of the concomitant symptomatology.

## Methods

### Study Design

Single centre, randomized, controlled study of two Physical Complex Therapies. The total follow-up period per patient is 6 months.

### Sample Size

For the calculation of the sample size we make the following assumptions:

• The effect of the standard treatment on the control group will cause an average limb volume reduction of 5%.

• The effect of the treatment on the experimental group will cause an average limb volume reduction of 25%

• We also assume that the standard deviation will be similar in both groups and near to 25%. With this information and with an alpha risk of 0.05 and a power of 0.80, the calculation of the sample size is 20 patients in each group. Considering a 30% dropout rate in each group, the corrected sample size is 58 patients randomized in two groups of 29 patients each.

### Subjects of study

Caucasian women with lymphoedema who have undergone a prior mastectomy, tumorectomy or quadrantectomy and axillary lymphadenectomy. A sample size of 58 patients randomized in 1:1 ratio (29 per group) will be required.

### Inclusion criteria

• Women older than 18 years treated for unilateral breast cancer with ipsilateral axillary lymphadenectomy.

• Patients with ipsilateral lymphoedema with a limb volume difference of at least 200 ml compared to the lateral member.

• Patients who have finished treatment with radiotherapy and/or chemotherapy at least six months before the study begins.

• Informed consent must be signed

### Exclusion criteria

• Bilateral arm involvement.

• Malignant Lymphoedema of acute appearance (within the first three months postintervention)

• Patients with paralysis or previous vascular disorder in the affected arm.

• Patients with a major limitation of 30% or more in any of the arcs of movement of the ipsilateral shoulder.

• Patients with a contraindication for Manual Lymphatic Drainage (cellulite, deep venous thrombosis, heart failure, uncontrolled hypertension, renal impairment and radiodermatitis.

• Patients who have undergone rehabilitation within three months prior to recruitment.

### Treatment groups

#### Group A or Control Group

Ambulatory treatment is performed for one month. Specific exercises and prevention measures are taught. In the first four weeks, multilayer bandage is applied daily. Bandaging consists of a first layer of cotton (Velband) followed by protection at the points of support and a bandage of low elasticity (Comprilan) of different widths (six centimeters for the area of the hand and eight centimeters for the rest of the extremity).

The tailor-made sleeve for lymphoedema with a gauntlet without protection at the edges and with extension to the shoulder is applied for the first four weeks of treatment. The sleeve is used all day, and at night interruption is allowed. Later on, the patient will continue domiciliary treatment, performing specific exercises for 30 minutes twice a day without experiencing fatigue, and wearing the lymphoedema sleeve for at least 12 hours.

If after three months of treatment a satisfactory response is not achieved, an ambulatory treatment will be re-established for one month, and this time the experimental treatment (group B) will be used.

#### - Group B. Experimental group

Ambulatory treatment is carried out for one month. Specific exercises and prevention measures are taught. In the first four weeks, MLD is applied followed by a daily multilayer bandage. The bandage consists of a first layer of cotton (Velband) followed by protection at the points of support and a bandage of low elasticity (Comprilan) of different widths (six centimeters for the area of the hand and eight centimeters for the rest of the extremity). The tailor-made sleeve for lymphoedema with a gauntlet without protection at the edges and with extension to the shoulder is applied for the first four weeks of treatment. The sleeve is used all day, and at night interruption is allowed. Later on, the patient will continue domiciliary treatment, performing specific exercises for 30 minutes twice a day without experiencing fatigue, and wearing the lymphoedema sleeve for at least 12 hours.

If after three months of treatment a satisfactory response is not achieved, an ambulatory treatment will be re-established for one month, and this time the treatment corresponding to group A or control group will be applied.

### Outcomes

#### Primary Outcome

Volume reduction of lymphoedema in the affected arm after treatment, expressed as a percentage. The condition is considered to be lymphoedema when there is a difference of at least 200 ml between the volume of the affected arm (measured by circometry and calculated using the formula of the truncated cone) and the volume of the contralateral arm. A response is considered to be good when a reduction of at least 20% of the volume is achieved after the treatment of lymphoedema in the affected extremity compared to baseline. These measurements will be performed at the beginning of the study, after the first and third month and after six months of follow-up. The volume difference has been used for the predetermination of the sample size.

#### Secondary Outcomes

The results of the Spanish validated quality of life questionnaires, EORTC QLQ-C30 version 2.0 for cancer in general and EORTC QLQ-BR23 specific for breast cancer, are used to assess the efficacy of the treatment, measuring the improvement of the lymphoedema concomitant symptomatology. Particularly, the items of the latter referring to the upper extremity (47, 48, 49) and to the corporal image (39, 40, 41, 42) are analyzed and the higher the score, the worse the result.

The secondary outcomes include:

• Degree of pain in the affected upper extremity

• Degree of sensation of swelling in the affected upper extremity

• Degree of functional limitation in this extremity

• Subjective feeling of being physically less atractive

• Subjective feeling of being less feminine

• Difficulty looking at oneself naked

• Dissatisfaction with the corporal image

### Statistical analysis

All patients included in the study will be analyzed and all the analyses will be performed in the "intention to treat" population, except those related to the withdrawal of treatment, which will be performed in the per-protocol population.

#### Statistical methods

Firstly, an analysis of the comparability of the groups will be performed using the Student-s T-test or Mann-Withney's Test and test of Chi^2 ^or Fischer's exact test. Subsequently, the effects found in the patients of the experimental group and the control group will be described using the difference of averages or ranges and risk ratios. These descriptions will be accompanied by their respective confidence intervals and hypothesis tests (as the ones mentioned above). When the result of the first analysis makes it necessary, possible confusion or the presence of interaction will be controlled using linear and logistic regression models.

#### Efficacy analyses

The analyses of the efficacy will be carried out using common measures such as mean and/or median ratio differences for the quantitative variables and risk ratios (relative risk) and NNT for the quantitative variables.

In all cases, the corresponding confidence intervals will be calculated.

##### Safety Analyses

Side effects, withdrawal and/or complication rates will be studied in the same way as those described for the quantification of the efficacy in the case of qualitative variables. In all case analyses, the confidence intervals will be also calculated.

##### Quality of Life Analyses

These analyses are carried out using the results of the Spanish validated quality of life questionnaire, EORTC QLQ-C30 version 2.0 for cancer in general, and EORTC QLQ-BR23 breast cancer-specific questionnaire, and they are assessed by the outcomes that the EORTC (European Organization for Research and Treatment of Cancer) allows.

### Subject's Data Protection

The study will strictly comply with the Declaration of Helsinki and the Spanish Data Protection Law (Constitutional law 15/1999 on Protection of Personal Data) and will protect at all times the patients' rights (Law 15/2002). The informed consent from each patient will be obtained and a randomized number will be assigned in order to protect the patient's anonymity.

### Interventions and distribution of tasks

The rehabilitation physician's functions include:

1. Selection call: the physician determines whether the patient meets inclusion criteria and if so, she is randomized into a treatment group. He/she carries out a complete medical history (history and complete physical exam, taking measurements of both upper extremities for the calculation of lymphoedema volume). He/she explains and provides the informed consent and collects the signed copy. He/she gives the quality of life questionnaire to the patient, who will complete it before starting the outpatient treatment.

2. Week 2 of treatment: Examine the patient. Take measurements of the upper extremities. Rule out the existence of treatment-related side effects. Make the request for a lymphoedema garment. Collect the quality of life questionnaires.

3. Week 5 of treatment: Examine the patient. Take measurements of the upper extremities. Rule out the existence of treatment-related side effects. Check the sleeve. Give a compliance diary to the patient.

4. Week 12 of treatment: To examine Explore the patient. Ask about possible complications that may lead to withdrawal from the study. Take measurements of both upper extremities. If the response to the treatment is good, the patient will be encouraged to continue complying with treatment. The patient's diary is reviewed. A second quality of life questionnaire is provided. The treatment is continued at home. If the response to the treatment is poor, the patient is included again in an outpatient treatment for a month so that if the patient has previously received TH now he/she would be treated with TH + DL and if he/she has been previously treated with TH + DL now she would be treated with DL. The patient is examined at week 16. Measurements of the upper extremities are taken. The presence of treatment-related side effects is ruled out.

5. Week 18 of treatment: the patient is examined. Measurements of the upper extremities are taken. The presence of treatment-related side effects is ruled out. The sleeve is reviewed. A second quality of life questionnaire is delivered.

6. Final call: Week 24 of treatment: the patient is examined. The patient is asked about possible complications that may lead to withdrawal from the study. Measures are taken in both upper limbs. The sleeve is reviewed.

At the end of the study, the patient's calendar is collected. The completed Quality of Life questionnaire is collected. Collaboration with the statistics department is carried out for data completion and publication.

The Physiotherapist's tasks include

1. She/he phones the patients of the study and notifies them of the starting of the treatment. She/he randomizes the patients in treatment A or B according to the instructions of the Biomedical Research Foundation.

2. She/he applies the prescribed treatment and instructs patients in the proper execution of exercise and care of the affected limb.

3. She/he makes the appointment with the patients for review in consultation.

4. She/he notifies the physician of any incidents that may occur.

Finally, the BIOMEDICAL RESEARCH FOUNDATION (BRF) randomizes the patients in the two different treatment groups, monitors the study and facilitates statistical analysis of the data and results.

### Ethical Approval

The study was approved by the Hospital Ethical Committee (Comite Ético de Investigación Clínica (CEIC) FIB Hospital Universitario de la Princesa) and will be conducted in accordance with the declaration of Helsinki.

## Discussion

### Relevance of the project in terms of its impact in clinical care

Within the Complex Physical Therapy (CPT), it is relevant to know how useful the Manual Lymph Drainage is because of the exclusive dedication of the therapist and the number of sessions required to complete this portion of the treatment. If it is proven to be effective, more staff trained in this technique should be provided to health centres so that affected patients can have access to it easily.

### Relevance of the project in terms of its bibliometric impact

The systematic review of literature on the management of lymphoedema was limited by the lack of prospective randomized trials evaluating different treatment options. In 2006, a Cochrane systematic review about physical treatments for reducing and controlling lymphoedema of the extremities selects only three of the articles reviewed on the basis that they are randomized controlled trials that had a follow-up period of at least six months. However, because they did not study the same treatment intervention, the data could not be subsequently pooled. Some evidence suggests that treatment involving a combination of MLD and compression therapy yields reduced oedema volume compared to compression therapy alone if volume is measured directly after the conclusion of MLD (Evidence grade 3 = limited scientific evidence). The existing randomized clinical trials are conducted with small samples and only a few studies include long term monitorization. None of the studies performed are blind and only rarely is an analysis of the rate of "lost to follow-up" patients performed. Before this therapy can be recommended, more randomized controlled trials with an adequate sample size, where MLD short-term and long-term treatment effects could be more closely studied, are needed (SBU Alert Report 2005-04. http://www.SBU.SE/ALERT).

Moreover, groups of experts have highlighted the lack of interest in researching the impact of the physical and psychosocial difficulties of these patients and their quality of life. In conclusion, the need for performing well-designed, randomized trials of the physical treatments and their health impact is a priority.

## Competing interests

The authors declare that they have no competing interests.

## Authors' contributions

MLM and MAH, physicians specialized in Physical Medicine and Rehabilitation, contributed with the initial approach of the idea and literature review. MLM is the main researcher of the study carrying out the selection of the patients, follow-up and data collection. The clinical trial monitor, CA, contributed with the adaptation and preparation of the protocol. FR was responsible for the statistical analysis and publishing tips. The physiotherapist, HM implemented the treatment to patients according to randomization, maintained contact with patients and reported incidents in the application of the treatment.

## Pre-publication history

The pre-publication history for this paper can be accessed here:

http://www.biomedcentral.com/1471-2407/11/94/prepub
